# Supplementary material for: Motor Imagery Training Is Beneficial for Motor Memory of Upper and Lower Limb Tasks in Very Old Adults
Source: Int J Environ Res Public Health. 2023 Feb 17;20(4):3541. doi: 10.3390/ijerph20043541 (PMC9963345; doi:10.3390/ijerph20043541)
Supplement: Supplementary file 1 [file ijerph-20-03541-s001.zip › ijerph-2175571-supplementary.pdf]

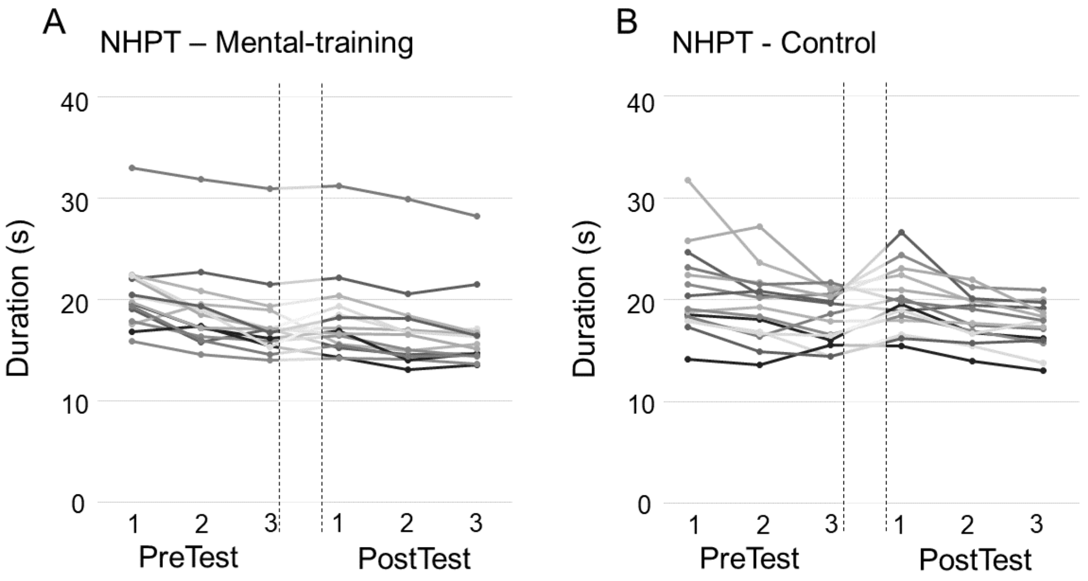

Figure S1: Individual trajectories across the 3 PreTest and PostTest trials for the mental-training (A) and control (B) groups, in the NHPT task.

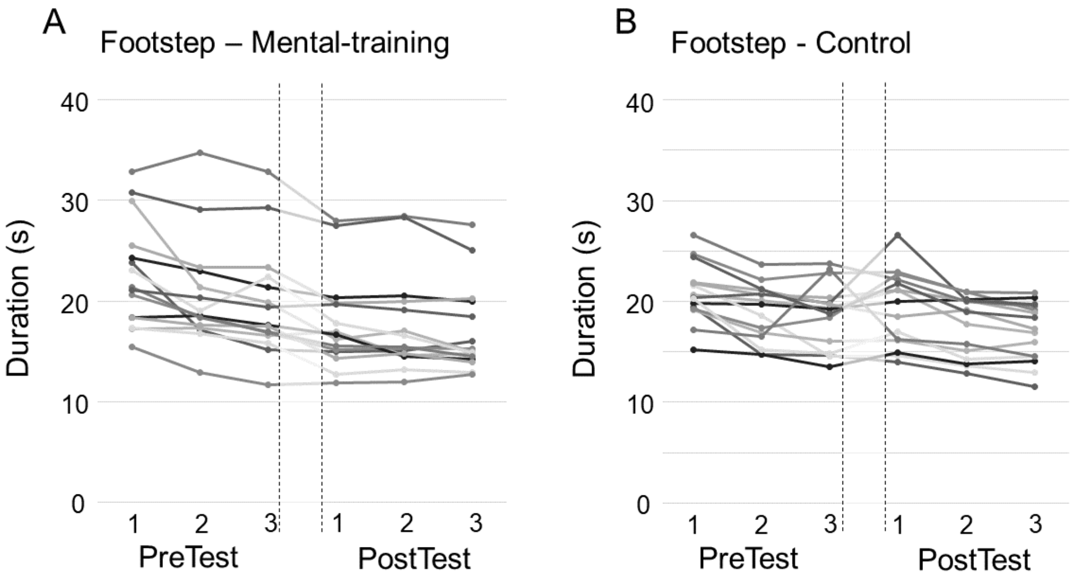

Figure S2: Individual trajectories across the 3 PreTest and PostTest trials for the mental-training (A) and control (B) groups, in the Footstep task.
